# Quercetin inhibition of porcine intestinal alpha coronavirus in vitro and in vivo

**DOI:** 10.1186/s12917-024-03984-2

**Published:** 2024-04-03

**Authors:** Yongzhi Feng, Heyou Yi, Xiaoyu Zheng, Xing Liu, Ting Gong, Dongdong Wu, Zebu Song, Zezhong Zheng

**Affiliations:** 1 Guangdong Provincial Key Laboratory of Zoonosis Prevention and Control, College of Veterinary Medicine, South China Agricultural University, Guangzhou, 510642 China; 2 Maoming Branch, Guangdong Laboratory for Lingnan Modern Agriculture, Maoming, 525000 China; 3 Key Laboratory of Animal Vaccine Development, Ministry of Agriculture and Rural Affairs, Guangzhou, 510000 China; 4 National Engineering Research Center for Breeding Swine Industry, South China Agricultural University, Guangzhou, PR China

**Keywords:** SADS-CoV, Quercetin, P53, Cell cycle, Antiviral activity

## Abstract

**Background:**

Porcine acute diarrhea syndrome coronavirus (SADS-CoV) is one of the novel pathogens responsible for piglet diarrhea, contributing to substantial economic losses in the farming sector. The broad host range of SADS-CoV raises concerns regarding its potential for cross-species transmission. Currently, there are no effective means of preventing or treating SADS-CoV infection, underscoring the urgent need for identifying efficient antiviral drugs. This study focuses on evaluating quercetin as an antiviral agent against SADS-CoV.

**Results:**

In vitro experiments showed that quercetin inhibited SADS-CoV proliferation in a concentration-dependent manner, targeting the adsorption and replication stages of the viral life cycle. Furthermore, quercetin disrupts the regulation of the P53 gene by the virus and inhibits host cell cycle progression induced by SADS-CoV infection. In vivo experiments revealed that quercetin effectively alleviated the clinical symptoms and intestinal pathological damage caused by SADS-CoV-infected piglets, leading to reduced expression levels of inflammatory factors such as TLR3, IL-6, IL-8, and TNF-α.

**Conclusions:**

Therefore, this study provides compelling evidence that quercetin has great potential and promising applications for anti- SADS-CoV action.

**Supplementary Information:**

The online version contains supplementary material available at 10.1186/s12917-024-03984-2.

## Background

Porcine enteric A coronavirus (PEAV), also known as porcine acute diarrhea syndrome coronavirus (SADS-CoV), was initially isolated from a diarrhea outbreak in a Guangdong, China-based herd in 2017. Two years later, the virus reemerged in an outbreak, resulting in economic losses for the local farming industry [1, 2]. Similar to other coronaviruses, SADS-CoV is a single-stranded, enveloped, positive-sense RNA virus with a genome of approximately 27 kb. It contains the 5’UTR, ORF1a/1b, S, NS3, E, M, N, NS7a, and 3’UTR [3]. Notably, SADS-CoV has the smallest S protein compared to other CoVs, with only 1,129 amino acids [1]. S proteins are recognized for their pivotal roles in virus attachment, cell entry, and antibody induction, making them significant targets against CoV infections [4]. Clinical symptoms in piglets caused by SADS-CoV, including diarrhea, dehydration, vomiting, and a high mortality rate, resemble those induced by other porcine intestinal pathogens [5]. Given the high mortality rate, there is a greater demand for effective antiviral drugs.

The limited transmission and small area of influence of SADS-CoV, a novel porcine enteric coronavirus, have resulted in fewer studies on related antiviral drugs. In our previous study, we found that DGAT-1 and metformin hydrochloride inhibited SADS-CoV replication in vitro by inhibiting lipid droplet formation [6]. Aloe vera rhodopsin exerted its antiviral effect by blocking SADS-CoV adsorption and the activation of the TLR3-IFN-λ3-ISG15 pathway [7]. Screening compound libraries revealed that cefpurine and methylene blue hindered SADS-CoV entry, while gemcitabine, mycophenolate mofetil, mycophenolic acid, and methylene blue inhibited replication post-viral entry [8]. Herbal medicines, being natural products, are widely available, exhibit low cytotoxicity, and have demonstrated significant efficacy in viral infectious diseases. Therefore, the search for new herbal medicines with anti- SADS-CoV potential is imperative.

Quercetin, a flavonoid found in fruits, vegetables, and medicinal plants, has attracted attention for its antioxidant, anti-inflammatory, antibacterial and anticancer properties [9]. It has been used as a food additive in some functional foods to prevent various diseases [10]. Quercetin has shown correlations with a wide range of viral infections, including Flaviviridae, Herpesviridae, Coronaviridae, and Orthomyxoviridae. Its antiviral effects encompass blocking the viral entry, promoting apoptosis, regulating the cell cycle, and inhibiting the expression of inflammatory factors [11]. Notably, the vast majority of quercetin can be absorbed by intestinal cells in glycoside form, suggesting a potential role in combating viral infections targeting the intestinal tract [12]. However, no in vivo or in vitro studies on the inhibition of SADS-CoV infection by quercetin have been reported.

There are no antiviral studies on quercetin for SADS-CoV. The aim of this study was to investigate the effect of quercetin on viral infections in vivo and in vitro, and to provide some theoretical basis for the treatment of SADS-CoV infections in the future.

## Materials and methods

### Quercetin-related target prediction

Compound targets were predicted using the PubChem database (https://pubchem.ncbi.nlm.nih.gov), the Swiss Target Prediction database (http://www.swisstargetprediction.ch/), and the PharmMapper database (http://www.lilab-ecust.co.uk/pharmmapper/index.html). The UniProt database (https://www.uniprot.org/) was used to convert all predicted target genes to gene names. Data from different databases were merged, and duplicates were removed.

### Diarrhea-related target prediction

Disease targets were predicted using the GeneCards database (https://www.genecards.org/), with “diarrhea” as the keyword.

### Cross-cutting targeting

The intersection of compound prediction targets with diarrhea prediction targets was determined using Venny2.1 (http://bioifogp.cnb.csic.es/tools/venny/index.html).

### Cells and viruses

IPI-FX cells (China Center for Type Culture Collection, CTCC, China) were cultured in Dulbecco’s Modified Eagle Medium (Gibco) containing 10% inactivated fetal bovine serum (BI) and 100 U/mL penicillin, 100 mg/mL streptomycin, and 25 µg/mL amphotericin. The viral infection and maintenance medium were serum-free DMEM containing 3‰ trypsin. Cells were incubated in an atmosphere of 5% CO2 at 37 °C. The SADS-CoVs were maintained by the Department of Infectious Diseases, College of Veterinary Medicine, South China Agricultural University. Piglets purchased from a farm.

### Cytotoxicity assay

The cytotoxicity of quercetin to cells was determined using the Cell Viability Counting Kit-8 (CCK-8; NCM Biotech, Suzhou, China) following the manufacturer’s instructions. Quercetin (PUSH BIO-TECHNOLOGY, Chengdu, China) was dissolved in DMSO at a concentration of 1 M. IPI-FX cells, grown to 90% confluence in 96-well plates, were treated with different drug concentrations, normal medium, or 0.1% DMSO. After 48 h, cells were washed thrice with PBS, and 100 µl of medium containing 10% CCK-8 was added to each well. The reaction proceeded at 37 °C for 30 min, and absorbance was measured at 452 nm using an enzyme meter.

### Indirect immunofluorescence

Cells were fixed at room temperature for 20 min using 4% paraformaldehyde, permeabilized with 0.3% TritonX-100 for 30 min, and blocked with 5% BSA for 1 h. Primary antibodies, anti- SADS-CoV N protein (1:500), were applied, followed by incubation with Alexa Fluor® 488 goat anti-mouse fluorescent secondary antibodies. After three washes with PBS, cell nuclei were restained with 4,6-di amidino-2-phenylindole (DAPI) (Beyotime, Shanghai, China). Fluorescence was observed using a Nikon DMI 4000b fluorescence microscope (Tokyo, Japan).

### Protein immunoblots

Cell samples were collected and lysed using RIPA lysate (Beyotime, Shanghai, China) containing 1% inhibitor. The supernatant was collected after centrifugation at 4 °C. Protein concentration was determined using a BSA Protein Concentration Measurement Kit (Beyotime, Shanghai, China) to ensure consistent sample volumes. The supernatants were boiled in 5× sodium dodecyl sulfate (SDS) sample buffer (Dingguo, Beijing, China). Protein samples were separated by electrophoresis on 10% SDS-polyacrylamide gels (Vazyme, Shanghai, China) and transferred onto polypropylene cellulose (NC) membranes (Merck, USA). The membrane was blocked for 1 h at room temperature with 5% skimmed milk powder (BD Difco, USA) and then incubated overnight at 4 °C using anti- SADS-CoV N protein (1:500) and GAPDH (Abmart, Shanghai, China) as primary antibodies. Proteins were incubated for 45 min with IRDye® 800CW goat anti-mouse IgG secondary antibody (LI-COR, USA) as the secondary antibody, washed thrice with TBST, and analyzed using a Sapphire RGBNIR Biomolecular Imager (Azure Biosystems, USA) imaging system.

### RNA extraction and real-time fluorescence quantitative PCR

Total RNA was extracted from IPI-FX cells using a Fastagen RNA extraction kit (Feijie, Shanghai, China). RNA was reverse-transcribed to cDNA using an RT-PCR kit (GenStar, Beijing, China). Real-time fluorescence quantitative PCR was performed using ChamQ SYBR qPCR Master Mix (Vazyme, Nanjing, China) according to the manufacturer’s instructions. The relative gene expression was calculated using the 2^−ΔΔCT^ method with GAPDH as the reference gene. The primers are listed in Table 1.

**Table 1:** Primers used for RT-qPCR

Primer	Sequence (5’→3’)
SADS-CoV N-F	CTGACTGTTGTTGAGGTTAC
SADS-CoV N-R	TCTGCCAAAGCTTGTTTAAC
TLR3-F	AATCATTACCAATCACACTTAAGCTGTTA
TLR3-R	CAAAACCAGCAACACGACTTTC
**IL6-F**	TGAACTCCTTCTCCACAAGC
IL6-R	GCGGCTACATCTTTGGAATC
IL8-F	CTGGCGGTGGCTCTCTTC
IL8-R	CCTTGGCAAAACTGCACCTT
TNF-α-F	GCCACCACGCTCTTCTGTCTG
TNF-α-R	AGGGGTCCTTGGGGAACTCTT
P53-F	CGCTTCGAGATGTTCCGAGA
P53-R	TTCAGGTGGCTGGAGTG
GAPDH-F	CCTTCCGTGTCCCTACTGCCAAC
GAPDH-R	GACGCCTGCTTCACCACCTTCT

### Inhibition of SADS-CoV infection assay

IPI-FX cells were inoculated in 12-well plates and incubated overnight. SADS-CoV with an MOI of 0.1 was incubated with different quercetin concentrations, 0.1% DMSO, or normal medium for 1 h at 4 °C, followed by three PBS washes to remove unabsorbed viruses. Cells were then cultured in medium containing various quercetin concentrations, 0.1% DMSO, or normal maintenance medium. After 48 h, cells were freeze-thawed three times, and the total viral RNA was detected using RT-qPCR.

### Direct virucidal assay

SADS-CoV, at an MOI of 0.1, was mixed with different quercetin concentrations and incubated at 37 °C for 1 h. Cells were inoculated in 96-well plates by serial multiplicative dilution and incubated for four consecutive days. Viral infectivity was visualized by IFA, and viral titers were calculated using the Reed-Muench method.

### Prophylactic measurements

IPI-FX cells were inoculated into 12-well plates and cultured overnight. Cells were exposed to medium containing various quercetin concentrations, 0.1% DMSO, or normal medium for 1 h. Subsequently, the cells were infected with virus at an MOI of 0.1 for 1 h at 4 °C, washed three times with PBS, and then replaced with maintenance medium. Viral load assays were conducted 48 h later.

### Adsorption assay

IPI-FX cells were inoculated into 12-well plates and incubated overnight. SADS-CoV with an MOI of 0.1 was inoculated in cell plates with different quercetin concentrations, 0.1% DMSO, or normal medium at 4 °C. After 1 h, cells were washed thrice with PBS and replaced with maintenance medium. Cells were collected after 48 h for viral RNA determination using RT-qPCR.

### Access to measurement

IPI-FX cells were inoculated in 12-well plates and incubated overnight. SADS-CoV with an MOI of 0.1, was added to the cells at 4 °C for 1 h. After three washes with PBS, cells were incubated with varying quercetin concentrations in a maintenance medium at 37 °C for 1 h. After another three washes with PBS, cells were incubated in fresh maintenance medium for 48 h. RNA was isolated from the cells for subsequent RT-qPCR to detect viral RNA content.

### Post-entry measurements

IPI-FX cells were seeded in 12-well plates and incubated overnight. SADS-CoV, with an MOI of 0.1, infected the cells at 4 °C for 1 h. Following three washes with PBS, maintenance medium was added. After 1 h, the maintenance medium was replaced with a maintenance medium containing varying quercetin concentrations. Cells were collected after 48 h, and viral RNA was quantified using RT-qPCR.

### Cell cycle analysis

SADS-CoV (0.1 MOI) infected cells were treated with 100 µM quercetin in maintenance medium after virus adsorption. After 48 h, cells were trypsinized, washed twice with PBS, and fixed with 70% anhydrous ethanol at -20 °C overnight. Subsequently, cells were collected by centrifugation, washed twice with pre-cooled PBS, and analyzed using propidium iodide (PI Beyotime, Shanghai, China) at 37 °C for 30 min. Samples were analyzed using a flow cytometer (Beckman Coulter, Miami, FL, USA).

### Animal experimentation

Two-day-old piglets were randomly divided into three groups of five piglets each: (1) Negative control group (mock), treated with diluent; (2) positive control group (SADS-CoV), SADS-CoV-infected and treated with a drug diluent; (3) treatment group (SADS-CoV + Quercetin), SADS-CoV-infected and treated with 10 mg/kg quercetin. In each group, 10 ml of the viral solution was orally administered to each pig, with a total amount tapping of 5 × 10^6^TCID50. The drug was administered orally every 12 h after tapping. Body temperature, weight, diarrhea, and piglet mortality were recorded, and anal swabs were collected for subsequent viral load determination. Daily diarrhea scores were assigned as follows: 0, normal; 1, mild; 2, moderate; 3, severe. The piglets were deeply anesthetized with phenobarbital in the ear vein, bled under anesthesia, and dissected after complete loss of vital signs. After the piglets’ death, intestinal tissues were collected to measure the viral load and inflammatory factor levels and were analyzed pathologically. Animal experiments were approved by the Animal Ethics Committee of South China Agricultural University (License No. 2023C007).

### Statistical analysis

Data analyses were performed using GraphPad Prism 8 software. The differences between groups were examined using a t-test. All experiments were performed in triplicate, and results were expressed as mean ± standard deviation (SD). Significance was indicated by *P* < 0.05.

## Results

### Quercetin and diarrhea biological target screening and PPI network construction

**Fig. 1:**
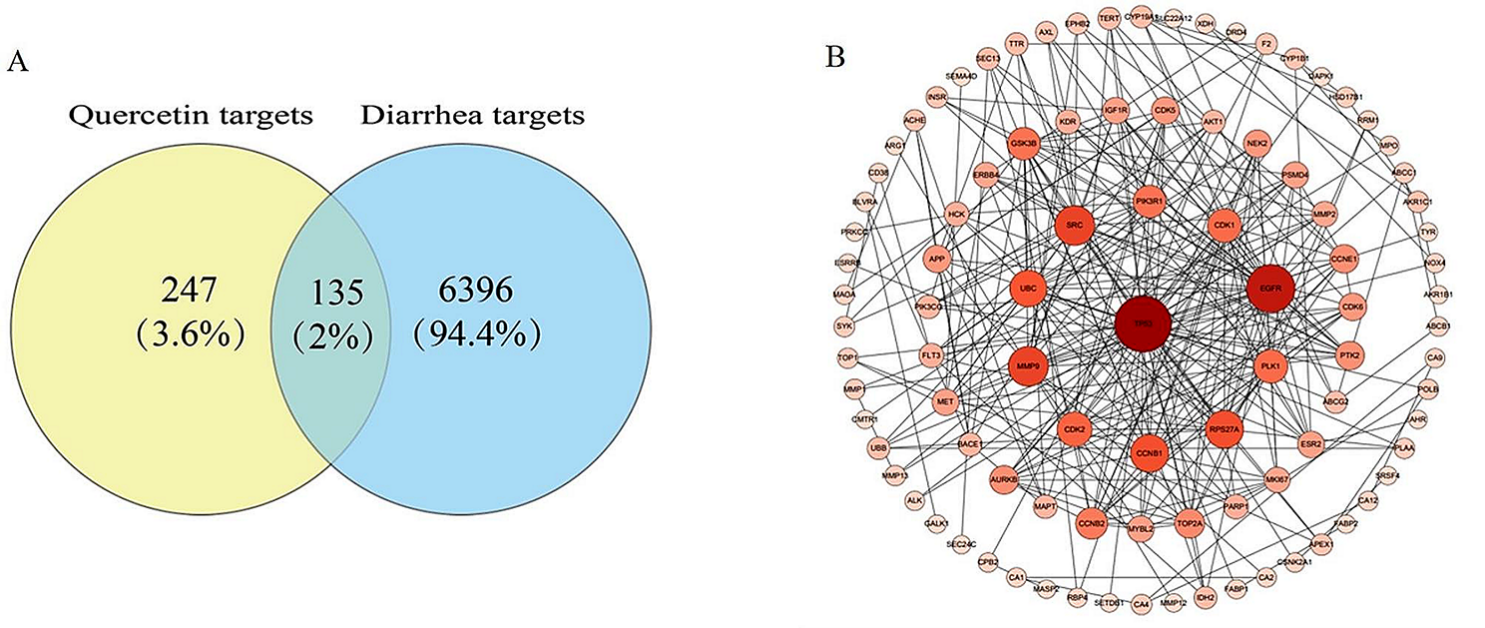
Quercetin active components and biological targets of diarrhea. (**A**) Venn diagram of quercetin and diarrhea. (**B**) PPI network constructed based on STRING database and Cytoscape

Based on the described methods, we identified 247 drug targets for quercetin and 6396 diarrhea-related targets. Among them, 135 targets overlapped (Fig. 1A). These 135 targets were uploaded to the STRING database to construct a PPI network using Cytoscape, and their nodes were rearranged based on degree value. Notably, P53 emerged as the primary potential target of quercetin (Fig. 1B).

### Quercetin inhibits SADS-CoV in vitro

**Fig. 2:**
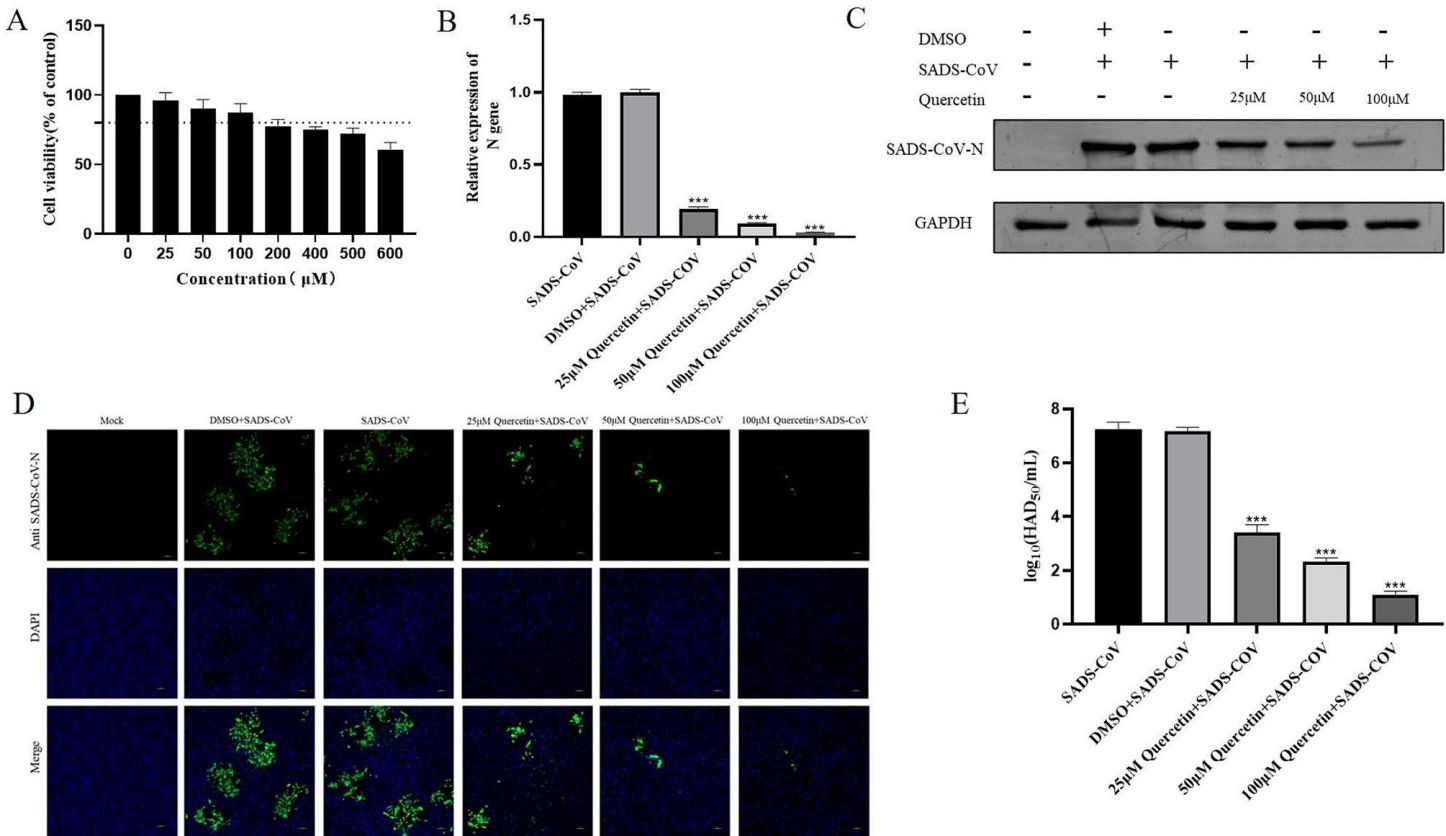
Anti- SADS-CoV activity of quercetin on IPI-FX. (**A**) IPI-FX was treated with different concentrations of quercetin for 24 h, and cell viability was measured by CCK-8 assay. (**B**) Infection with SADS-CoV (0.1 MOI) in the presence of different concentrations of quercetin was replaced with fresh medium containing different concentrations of quercetin supplemented with 3‰ trypsin after 1 h, and the relative SADS-CoV N mRNA levels were determined by RT-qPCR at 24 h, with DMSO as a treatment control. Results are from one of three independent experiments (*n* = 3), and asterisks in the graphs indicate significant differences (*P < 0.05; **P < 0.01; ***P < 0.001; ns: not significant). (**C**) Cell lysates were collected at 24 h for protein blot identification. (**D**) SADS-CoV N protein content was detected by IFA under fluorescence microscope, SADS-CoV N protein was shown in green color and nuclei in blue color. (**E**) Release of quercetin-treated SADS-CoV into cell supernatants in daughter virus titers

To test the cytotoxicity of quercetin, IPI-FX cells were treated with varying quercetin concentrations for 48 h, and cell viability was evaluated using the CCK8 assay. No significant cytotoxicity was observed when quercetin concentrations remained below 100 µM (Fig. 2A). Consequently, subsequent antiviral assays employed concentrations of 25 µM, 50 µM, and 100 µM. Cells were infected with SADS-CoV (0.1 MOI) in the presence of quercetin (25 µM 50 µM, 100µM). The virus adsorption was replaced with medium containing different quercetin concentrations and cells and cell lysates were collected for SADS-CoV N mRNA and protein detection. Results showed a dose-dependent reduction in SADS-CoV N mRNA and protein levels with increasing quercetin concentrations (Fig. 2B-C). The raw image of Fig. 2C was shown in Supplementary Information file 1. IFA results indicated a gradual decrease in SADS-CoV content with increasing quercetin concentrations (Fig. 2D). Meanwhile, quercetin dose-dependently reduced the titer of progeny virus (Fig. 2E). These results underscore quercetin’s dose-dependent inhibition of SADS-CoV.

### Effect of quercetin on SADS-CoV inactivation, prevention, attachment, internalization, and replication

**Fig. 3:**
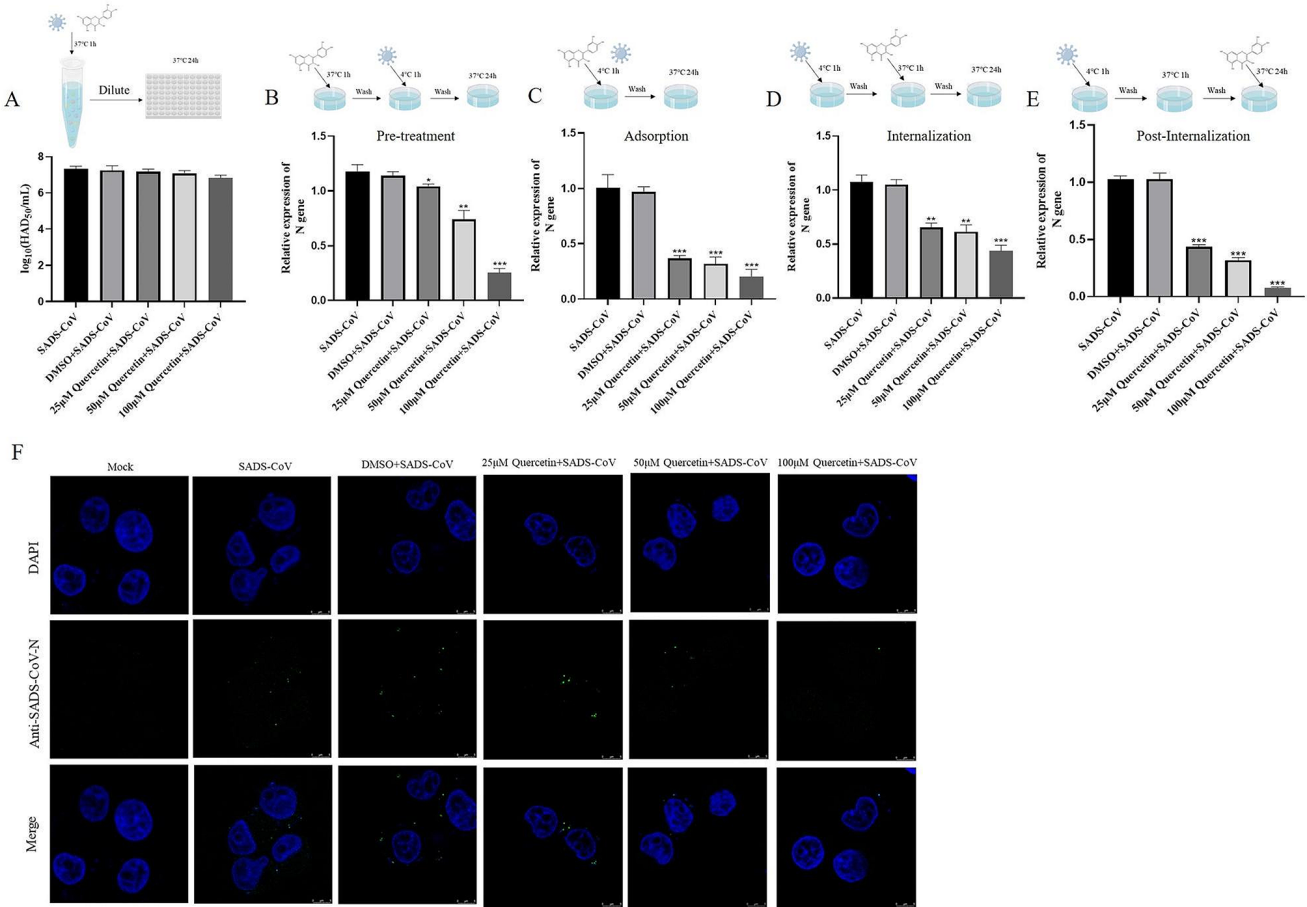
Effect of quercetin on SADS-CoV inactivation, adsorption, entry, and replication. (**A**) Virucidal assay. Different concentrations of quercetin were used to co-incubate with SADS-CoV (0.1 MOI) at 37 °C for 1 h, then diluted and inoculated in 96-well plates, and virus titers were measured by TCID50. (**B**) Virus prophylaxis assay. Different concentrations of quercetin were pretreated with IPI-FX for 1 h, and SADS-CoV (0.1 MOI) was adsorbed and replaced with maintenance medium. (**C**) Virus adsorption assay. Different concentrations of quercetin with SADS-CoV (0.1 MOI) were contacted with IPI-FX at 4 °C for 1 h and then replaced with maintenance medium. (**D**) Virus internalization assay. SADS-CoV (0.1 MOI) was adsorbed and then replaced with medium containing different concentrations of quercetin for 1 h and then replaced with maintenance medium. (**E**) Viral replication assay. SADS-CoV (after entry), replaced with maintenance medium containing different concentrations of quercetin. DMSO treatment was used as a control, and cell lysates were collected at 24 h for RT-qPCR, and SADS-CoV N content was calculated using 2^−ΔΔCT^. Results are from one of three independent experiments (*n* = 3) and asterisks in the graphs indicate significant differences (*P < 0.05; **P < 0.01; ***P < 0.001; ns: not significant). (**F**) Quercetin inhibits SADS-CoV adsorption. IPI-FX was incubated with SADS-CoV (5 MOI) or different concentrations of quercetin or DMSO at 4 °C for 1 h, and virus particle adsorption was observed by IFA

To determine the effect of quercetin on SADS-CoV infection, we first tested whether quercetin directly inactivates the virus. Results showed that quercetin, at different concentrations, did not directly inactivate SADS-CoV (Fig. 3A). Next, we added quercetin at different time points to determine its effects on SADS-CoV prevention, adsorption, internalization, and replication. Cell samples collected 24 h later were analyzed for SADS-CoV N mRNA levels. Quercetin exhibited inhibitory effects at all stages of the viral infection cycle, with the most significant impact observed during virus adsorption and replication (Fig. 3B-E). A virus adsorption assay employing IFA confirmed quercetin’s ability to reduce the number of viral particles adsorbed onto the cell surface (Fig. 3F). In summary, quercetin primarily inhibits SADS-CoV by affecting viral adsorption and internalization.

### Quercetin inhibits cell cycle progression accelerated by SADS-CoV infection

**Fig. 4:**
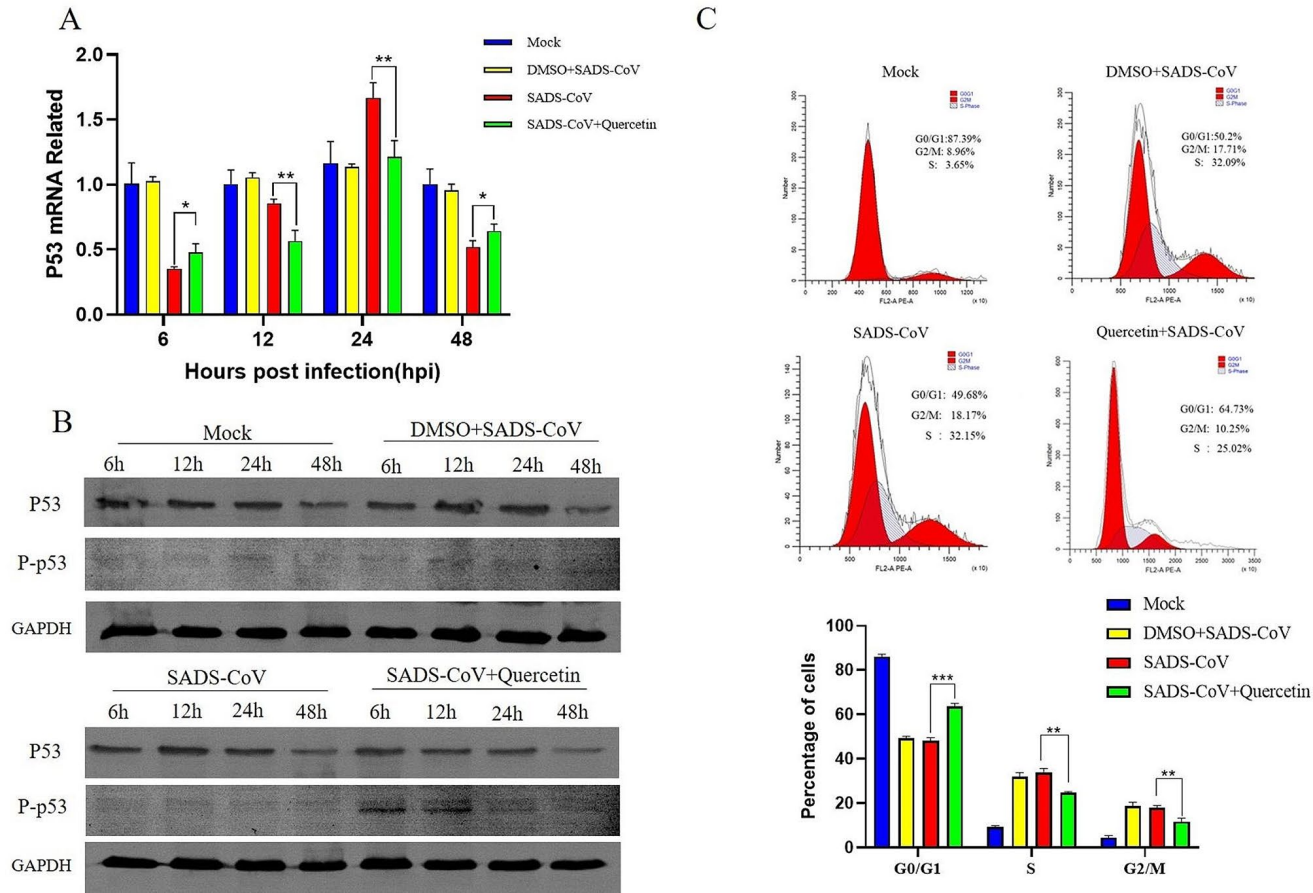
Quercetin regulation of P53 as well as cell cycle induced by SADS-CoV infection (**A**) Cell lysates were collected at different time points and total RNA was extracted, and the P53 gene mRNA profile was detected using RT-qPCR. (**B**) Effects of SADS-CoV and quercetin on P53 protein levels and phosphorylation levels. (**C**) Cells were collected to analyze cell cycle distribution by flow cytometry. Results are from one of three independent experiments (*n* = 3), and asterisks in the graphs indicate significant differences (*P < 0.05; **P < 0.01; ***P < 0.001; ns: not significant)

Given our analysis indicating that P53 plays a pivotal role in the pharmacological PPI network of quercetin, and considering that 100 µM quercetin significantly inhibited viral infection, we chose this concentration for subsequent experiments. Subsequently, we monitored changes in P53 transcript levels in cells after SADS-CoV infection. The results showed that SADS-CoV significantly inhibited P53 gene expression at 6 h, 12 h and 48 h post-infection, while promoting it at 24 h. Quercetin treatment successfully reversed the regulatory changes in P53 transcription induced by SADS-CoV infection (Fig. 4A). However, SADS-CoV infection and quercetin treatment did not significantly alter the protein level of p53, but quercetin promoted the phosphorylation of p53 at an early stage (Fig. 4B). The raw image of Fig. 4B was shown in Supplementary Information file 2. Notably, P53 is an important cycle-regulating protein [13]. Thus, we examined changes in the cell cycle after SADS-CoV infection, revealing that SADS-CoV accelerates cell cycle progression. Additionally, quercetin treatment suppressed SADS-CoV induced cell cycle changes (Fig. 4C). These results suggest that SADS-CoV infection regulates P53 transcript levels and accelerates cell cycle progression, which quercetin effectively retards.

### Therapeutic effect of quercetin on SADS-CoV -infected piglets

**Fig. 5:**
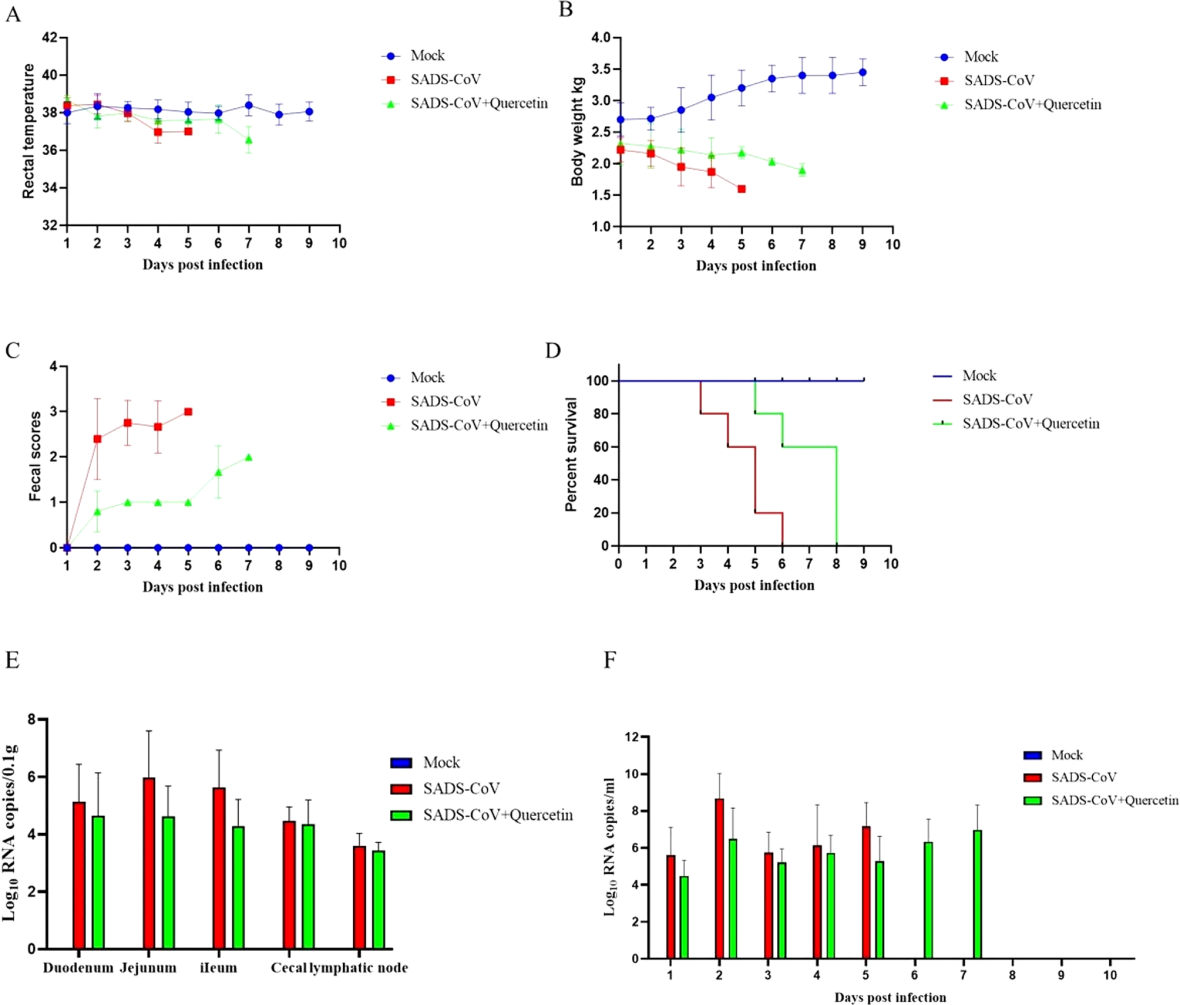
Therapeutic effect of quercetin on SADS-CoV infection. (**A**) Change in body temperature of piglets. (**B**) Changes in piglet weight. (**C**) Diarrhea of piglets. (**D**) Survival rate of piglets. (**E**) Viral load in the intestinal tract and mesenteric lymph nodes of pigs. (**F**) Fecal viral load. Intestinal and fecal viral loads were detected using RT-qPCR, and the results were from one of three independent experiments (*n* = 3), and the asterisks in the graphs indicate significant differences (*P < 0.05; **P < 0.01; ***P < 0.001; ns: not significant)

Following viral infection, the survival rate, body temperature, body weight, and diarrhea of the piglets were recorded daily. The results showed that after viral inoculation, the piglets exhibited clinical symptoms such as diarrhea, vomiting, and weight loss on the 2nd day, In the virus-infected group, mortality began on the 3rd day, with all five pigs succumbing by the 6th day. In the treatment group, symptoms were milder during the first four days, but piglet mortality began on the 5th day, with all five pigs succumbing by the 8th day. Conversely, piglets in the mock group remained symptom-free throughout the experiment, with all surviving until the end of the experiment (Fig. 5A-D). These results indicated that quercetin significantly reduced the clinical symptoms caused by SADS-CoV infection. Furthermore, we collected fecal swabs, intestines, and mesenteric lymph nodes from the slaughtered piglets for viral load assessment using RT-qPCR. The results showed lower viral content in the intestines, lymph nodes, and feces of piglets in the treatment group compared to those in the virus-infected group (Fig. 5E-F). The results showed that the quercetin-treated group had an overall lower viral load than the SADS-CoV-infected group.

### Gross pathology, histopathology

**Fig. 6:**
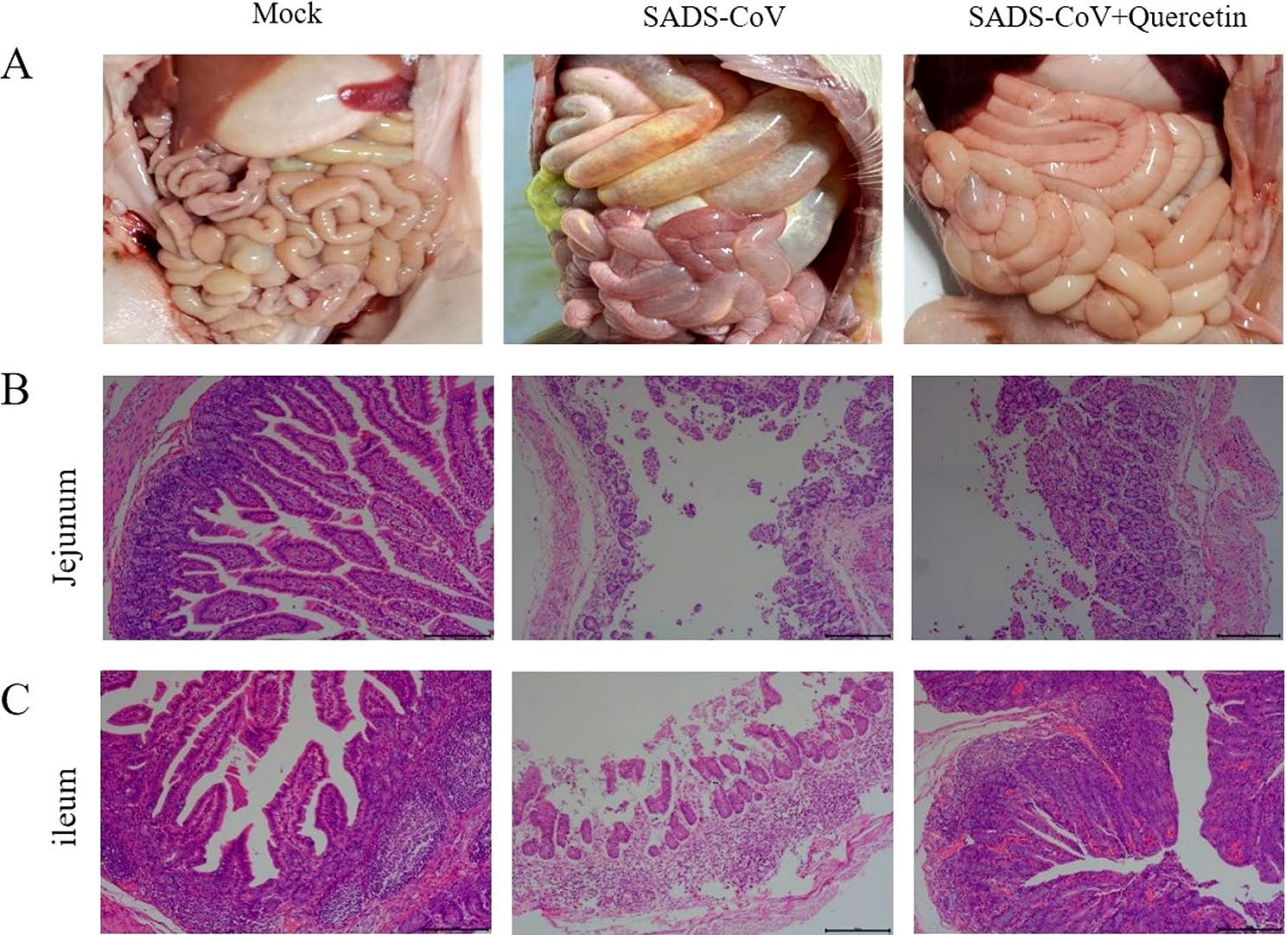
Intestinal dissection and microscopic observation of piglets. (**A**) Intestinal dissection of piglets in control, SADS-CoV -infected and drug-treated groups. (**B**) HE staining of jejunum. (**C**) HE staining of ileum

Following piglet dissection, their intestines underwent histopathological examination. The results showed that the intestines in the SADS-CoV-infected group were gas-filled and transparent, and these symptoms were reduced in the quercetin treated group (Fig. 6A). In addition, HE staining results demonstrated atrophied, broken, and detached intestinal villi in the SADS-CoV-infected group, whereas the treated group displayed relatively intact villi with clear outlines (Fig. 6B-C). These results indicate that quercetin attenuates intestinal damage caused by SADS-CoV infection.

### Inflammation of intestinal tissue

**Fig. 7:**
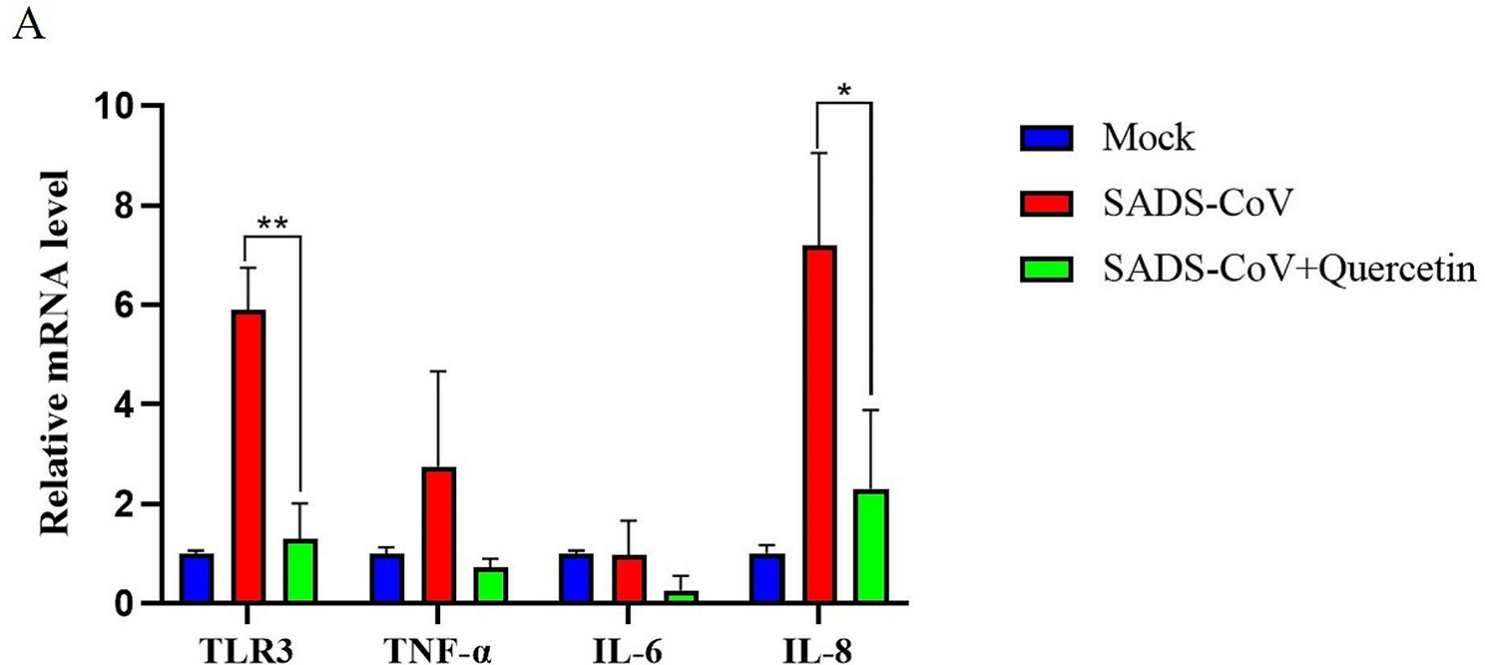
Inhibition of SADS-CoV infection-induced inflammatory factors by quercetin. (**A**) Piglet ileum was collected and mRNA expression of TLR3, TNF-α, IL-6, and IL-8 was detected in the tissue by RT-qPCR. Results are from one of three independent experiments (*n* = 3), and asterisks in the graphs indicate significant differences (*P < 0.05; **P < 0.01; ***P < 0.001; ns: not significant)

Toll-like receptor 3 (TLR3) recognizes viral RNA, activating the NF-κB pathway and inducing inflammatory cytokines expression. However, excessive inflammation can lead to organ failure and death [14]. Ileal tissue RNA extraction from all piglet groups revealed increased pro-inflammatory factor mRNA levels following SADS-CoV infection. Yet, levels of pro-inflammatory factors in quercetin treated piglets were significantly lower compared to the infected group (Fig. 7A). These findings indicate that quercetin could inhibit the up-regulated levels of TLR3, TNF-α, IL-6, and IL-8 pro-inflammatory factors induced by SADS-CoV infection, effectively reducing the associated inflammatory response.

## Discussion

SADS-CoV once posed a significant threat to the regional farming industry and exhibit zoonotic potential owing to their cytophilicity. The search for effective drugs is crucial for potential future outbreaks. Natural herbs have already played important roles in clinical practice, including curcumin, tretinoin, and ginseng saponins, which have been used in clinical cancer treatment [15]. The increased emphasis on food safety and the concept of “reducing anti-inflammatory drugs instead of anti-inflammatory drugs” have led to a growing use of Chinese herbs in aquaculture. These herbs are used as antimicrobials, immunoprophylactic agents, and food promoters. Some traditional Chinese herbs have also proven effective in enhancing the immunity, production performance, and product quality of chickens [16–18]. This study highlights the potential of natural flavonoids as antiviral agents and demonstrates that quercetin effectively inhibits SADS-CoV infection in vivo and in vitro, supporting its potential for prophylactic and adjuvant therapies.

The viral life cycle involves adsorption, entry, replication, and release, making interruption in this cycle an effective strategy for preventing and treating viral infections [19, 20]. For instance, Hypericin effectively prevents replication of PEDV, with limited impact on adsorption and entry [21]. In contrast, Aloe vera rhododendron and allicin demonstrate diverse effects on the life cycle phases of PRRSV, including direct virucidal activity [22, 23]. In the case of of African swine fever virus (ASFV), kaempferol, a flavonoid, affects the synthesis of viral proteins and DNA during adsorption and replication [24]. This study utilizing RT-qPCR, establishes that quercetin affects various stages of the SADS-CoV life cycle. Notably, quercetin does not directly inactivate SADS-CoV, suggesting the need for further investigation into its interaction with viral and host receptor proteins. In studies conducted by other groups, it was found that quercetin exhibits a dose-dependent inhibition of FMDV replication in vitro by inducing IFN production. It also demonstrates a significant antiviral effect during the early stages of FMDV entry and enhances the survival rate of FMDV-infected mice [25]; Moreover, quercetin has been shown to inhibit the replication of HCV and the production of infectious particles. It affects the integrity of viral particles, indicating a direct, host-mediated antiviral effect on HCV [26]; Similarly, quercetin has been observed to block BVDV infection at an early stage, inhibit BVDV replication, and ameliorate BVDV-induced histopathological damage by inhibiting oxidative stress or ERK phosphorylation [27]. These findings collectively suggest that quercetin holds promise as a broad-spectrum antiviral agent. Antiviral compounds are commonly designed to target either the virus itself or the host, and a range of host-targeted compounds have been identified to effectively inhibit viral entry and replication without inducing cytotoxicity [28]. Some compounds exert their effects by interfering with the glycosylation of cellular receptors, elevating the pH within endosomes to hinder virus-cell fusion, and inducing rearrangements of the cell membrane [29, 30]. In a study conducted by our group, it was observed that SADS-CoV can exploit multiple pathways for host cell invasion, with its entry being pH-dependent [3]. Investigating whether quercetin, along with other compounds, can modulate the invasion pathways of SADS-CoV and potentially alter intracellular pH represents a crucial direction for future research.

P53, a crucial transcription factor, responding to cellular stress, such as oxidative stress and metabolic changes, initiates regulatory processes including DNA damage repair, apoptosis, and cell cycle arrest [31]. The relationship between P53 and these processes is complex and not yet fully understood [32]. The cell cycle is one of the most fundamental processes in cell growth and division, and normal development and homeostasis require strict control over the cell cycle. The cell cycle usually consists of a gap 1 phase (G1), a DNA synthesis phase (S), a gap 2 phase (G2), and a mitotic phase (M); the cell cycle arrest phase after mitosis belongs to the G0 phase [33]. Many viruses have evolved different ways to regulate the cell cycle to facilitate their own infection process. For example, porcine circovirus type 2 (PCV2) promotes its replication by inducing host cells into the S phase, swine fever virus (CSFV) increases the expression of P53 to arrest the cell cycle in G1 during infection, and simian virus 40 (SV40) activates ATR-Delta P53 signaling, maintaining the host in the S phase to create an optimal environment for SV40 replication [34–36]. In our study, the transcriptional regulation of SADS-CoV on P53 varied with time. Quercetin affects the regulation of p53 transcription by SADS-CoV while promoting the level of p53 phosphorylation at an early stage and activating the antiviral response. However, the overall observation was a decrease in the host’s G0/G1 phase and an increase in the S phase and G2/M phase after SADS-CoV infection. This indicates that SADS-CoV accelerates the host cell cycle and that quercetin treatment inhibited virus-induced accumulation in the S and G2/M phases, retarding the cell cycle. While the above results demonstrate that quercetin can inhibit the acceleration of the host cell cycle by SADS-CoV, further experiments are needed to verify the specific effects of SADS-CoV on cycle regulation pathways at different time points, and the mechanism through which SADS-CoV regulates the cell cycle.

SADS-CoV infection typically causes vomiting, diarrhea, dehydration and death in piglets. The study administered 10 mg/kg quercetin orally to assess its practical application in clinical practice. The results showed that quercetin improved diarrhea, reduced the viral load in the intestinal tract and feces, and suppressed the expression of TLR3, TNFα, IL6, and IL8 pro-inflammatory factors induced by SADS-CoV. Although quercetin provided some protection to SADS-CoV-infected piglets, it did not ensure their continuous survival. This may be attributed to quercetin’s inability to reverse the intestinal damage caused by SADS-CoV infection, leading to nutrient loss and the eventual death of the piglets. In summary, quercetin, a natural product from various sources, significant inhibits SADS-CoV in vivo and in vitro, showing high potential as a prophylactic or adjuvant treatment for SADS-CoV-infected piglets.

## Conclusion

In this study, we observed dose-dependent antiviral effects of quercetin at different stages of SADS-CoV infection. Quercetin demonstrated the ability to inhibit SADS-CoV infection, thereby accelerating cell cycle progression. In vivo experiments revealed the efficacy of quercetin in alleviating clinical symptoms caused by SADS-CoV and reducing the expression levels of inflammatory factors such as TLR3, IL-6, IL-8, and TNF-α.

### Electronic supplementary material

Below is the link to the electronic supplementary material.

Supplementary Material 1

Supplementary Material 2

## Data Availability

No datasets were generated or analysed during the current study.

## Abbreviations

SADS-CoV: porcine acute diarrhea syndrome coronavirus
IFA: Indirect immunofluorescence
RT-qPCR: RNA extraction and real-time fluorescence quantitative PCR
