# Correction: Quercetin inhibition of porcine intestinal alpha coronavirus in vitro and in vivo

**DOI:** 10.1186/s12917-024-04031-w

**Published:** 2024-04-29

**Authors:** Yongzhi Feng, Heyou Yi, Xiaoyu Zheng, Xing Liu, Ting Gong, Dongdong Wu, Zebu Song, Zezhong Zheng

**Affiliations:** 1 Guangdong Provincial Key Laboratory of Zoonosis Prevention and Control, College of Veterinary Medicine, South China Agricultural University, Guangzhou, 510642 China; 2 Maoming Branch, Guangdong Laboratory for Lingnan Modern Agriculture, Maoming, 525000 China; 3 Key Laboratory of Animal Vaccine Development, Ministry of Agriculture and Rural Affairs, Guangzhou, 510000 China; 4 National Engineering Research Center for Breeding Swine Industry, South China Agricultural University, Guangzhou, PR China

**Correction:** ***BMC Vet Res*** **20, 134 (2024)**

10.1186/s12917-024-03984-2

Following publication of the original article [1], the authors would like to remove the author Qin Peng from the author list. The original article has been corrected.
