# Supplementary material for: Quercetin inhibition of porcine intestinal alpha coronavirus in vitro and in vivo
Source: BMC Vet Res. 2024 Apr 3;20:134. doi: 10.1186/s12917-024-03984-2 (PMC10988794; doi:10.1186/s12917-024-03984-2)
Supplement: Supplementary file 1 — Supplementary Material 1 [file 12917_2024_3984_MOESM1_ESM.pdf]

The uncropped Western blot shown in Fig.4B

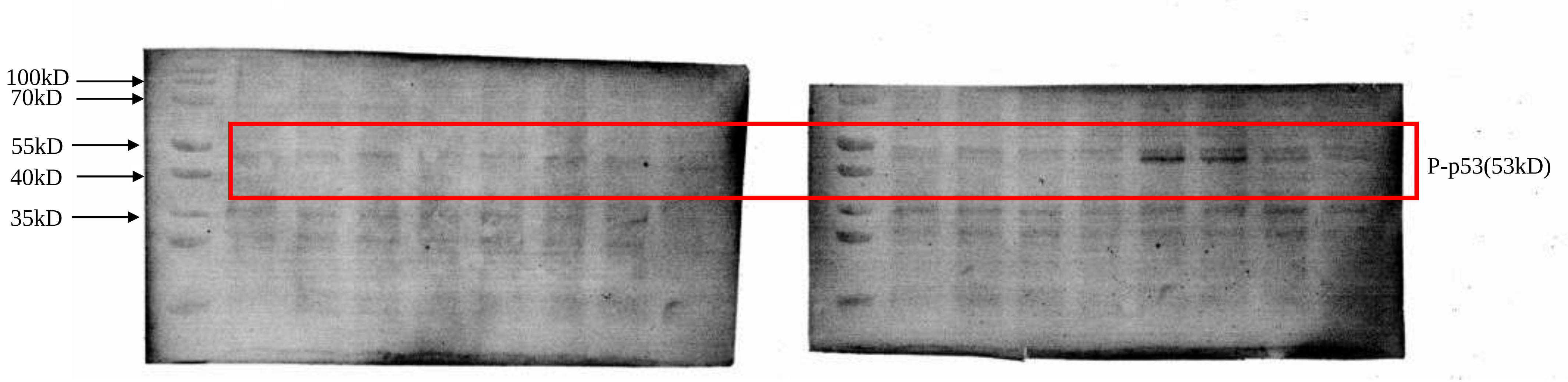

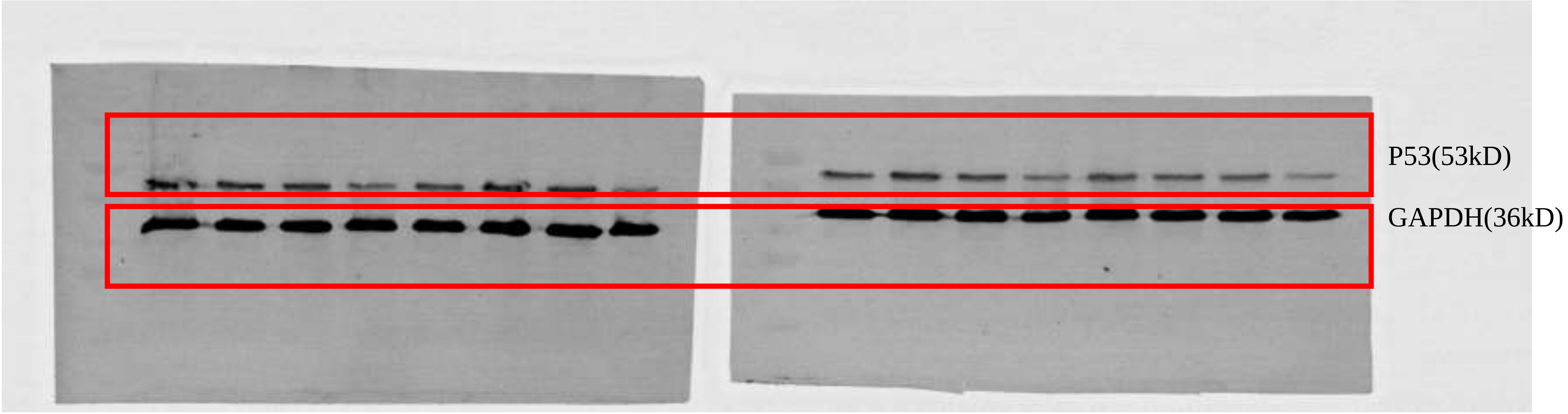

Figure S2 represents the uncropped western blot scan shown in Figure 4.
