# Supplementary material for: Quercetin inhibition of porcine intestinal alpha coronavirus in vitro and in vivo
Source: BMC Vet Res. 2024 Apr 3;20:134. doi: 10.1186/s12917-024-03984-2 (PMC10988794; doi:10.1186/s12917-024-03984-2)
Supplement: Supplementary file 2 — Supplementary Material 2 [file 12917_2024_3984_MOESM2_ESM.pdf]

The uncropped Western blot shown in Fig.2C

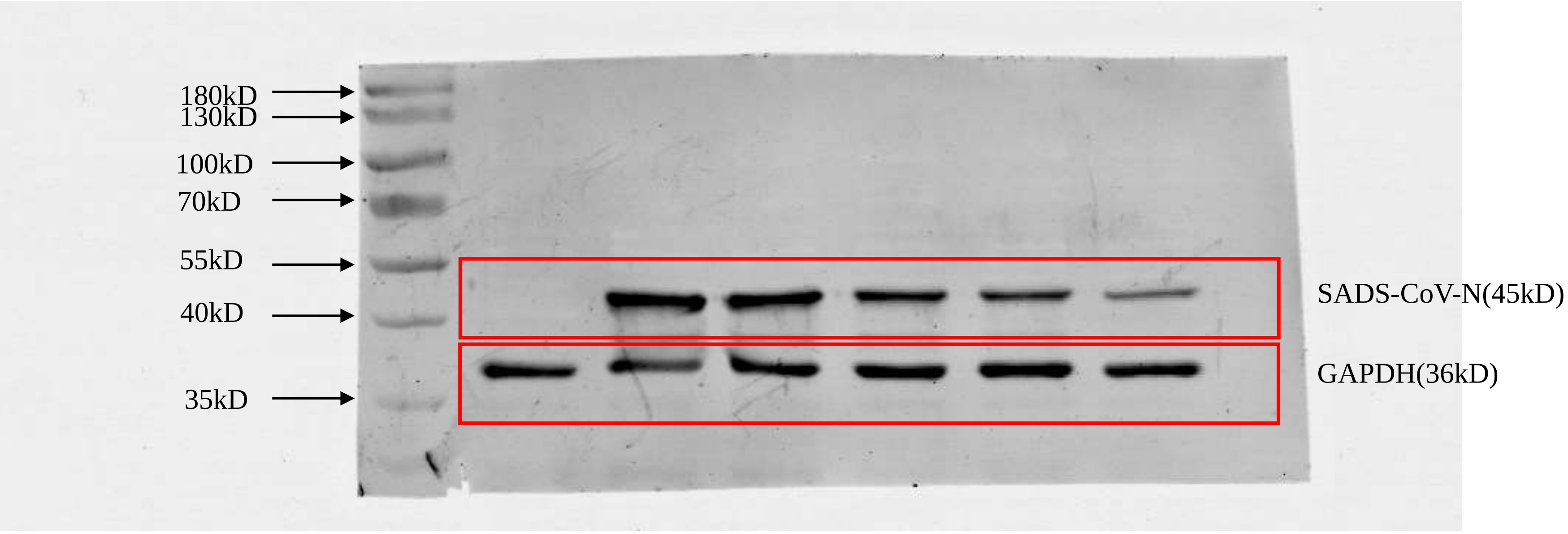

Figure S1 represents the uncropped western blot scan shown in Figure 2.
